# State of nature 2023 terrestrial and freshwater animal dataset for the United Kingdom and its constituent countries

**DOI:** 10.1016/j.dib.2025.111646

**Published:** 2025-05-12

**Authors:** Fiona Burns, Simone Mordue, Nida Al-Fulaij, Philipp Boersh-Supan, Katherine L. Boughey, Philip Briggs, Mark A. Eaton, Colin Harrower, Angus C. Jackson, Steve Langton, Francesca Mancini, David Noble, Chris R. Shortall, Chloë A. Smith, Robin J. Pakeman

**Affiliations:** a RSPB, Centre for Conservation Science, David Attenborough Building, Pembroke Street, Cambridge CB2 3QZ, United Kingdom; b People’s Trust for Endangered Species, 3 Cloisters House, 8 Battersea Park Road, London SW8 4BG, United Kingdom; c British Trust for Ornithology, Thetford IP24 2PU, United Kingdom; d Bat Conservation Trust, Studio 15 Cloisters House, Cloisters Business Centre, 8 Battersea Park Road, London SW8 4BG, United Kingdom; e ME Rare Breeding Bird Panel, Alnwick NE66 1EL, United Kingdom; f UK Centre for Ecology & Hydrology, Maclean Building, Benson Lane, Crowmarsh Gifford, Wallingford, Oxfordshire OX10 8BB, United Kingdom; g Seasearch, Marine Conservation Society, Overross House, Ross Park, Ross-on-Wye HR9 7US, United Kingdom; h Hallgarth, Leavening, Malton, N Yorkshire YO17 9SA, United Kingdom; i Rothamsted Insect Survey, Rothamsted Research, Harpenden AL5 2JQ, United Kingdom; j Butterfly Conservation, Manor Yard, East Lulworth, Wareham, Dorset BH20 5QP, United Kingdom; k The James Hutton Institute, Craigiebuckler, Aberdeen AB15 8QH, United Kingdom

**Keywords:** Abundance, Biodiversity, Long-term trend, Occupancy, Short-term trend

## Abstract

This article describes the terrestrial and freshwater animal trend data used in creating the 2023 State of Nature reports for the UK and its constituent countries. Trend data for long- (1970–2020/21) and short-term (2010–2020) periods have been calculated by fitting statistical models to measures of abundance (753 species) or occupancy (4979 species) across the UK. Trend data was also calculated for each constituent country: England, Northern Ireland, Scotland and Wales for reduced sets of species.

Trends in abundance data were generally created from the analysis of repeat counts at specific sites. Trends in occupancy were created by analysing ad hoc species records of invertebrates provided by volunteers. Statistical methods partially controlled for the risk of bias and the noisy nature of such occupancy data. Trends were only calculated where the number of species records justified the creation of trend statistics.

Species that make up three key groups of insects are identified in additional columns in the dataset, those responsible for key ecosystem functions: species providing freshwater nutrient cycling, pollinating insects and predators of crop pests.

The data has one clear limitation in that it is only a partial representation of the fauna of the UK. Many cryptic, nocturnal or soil dwelling species are poorly recorded and even some easy to identify species such as amphibians and reptiles do not have a suitable recording scheme that captures abundance.

Specifications Table

**Table:** 

Subject	Biology
Specific subject area	Species trend data at the UK and constituent country level.
Type of data	Analysed
Data collection	Trend data calculated by fitting statistical models to measures of abundance (753 species) or occupancy (4979 species) across the UK. Trend data is present for long- (1970–2020/21) and short-term (2010–2020) periods for a range of animal species for the whole of the UK, as well as for each constituent country: England, Northern Ireland, Scotland and Wales.
Data source location	All of terrestrial UK.
ta accessibility	Repository name: Zenodo Data identification number: 10.5281/zenodo.14209674 Direct URL to data: 10.5281/zenodo.14209674
Related research article	None

## 1 Value of the Data

• The State of Nature reports the most up-to-date information available for species trends in the United Kingdom and its constituent countries to give an overview of how biodiversity is faring nationally.
• Published reports provide headline analyses aimed at summarizing the data for policy makers and the public. These reports set out the numbers of species increasing in abundance or occupancy, those with little change and those decreasing, and then links these trends to the drivers of change, including conservation action.
• Providing species level trend information as an open data set will allow for wider participation in analysis and allow a more detailed identification of patterns in the data and likely drivers of species trends.

## 2 Background

State of Nature reports have been produced for the United Kingdom and its constituent countries in 2013, 2016, 2019 and 2023. They have been produced as a partnership between conservation NGOs, research institutes and universities, with the statutory conservation agencies joining as full signatories from 2019 onwards (66 organizations). The reports aim to provide a common evidence base on the state of biodiversity, the pressures it faces, the benefits of conservation action and to communicate this assessment widely and effectively to a broad suite of target audiences.

The 2023 reports [[1], [2], [3], [4], [5]] bring together the latest and best data from a wide range of biological monitoring and recording schemes. Several of the same datasets are also used to create a number of statutory biodiversity indicators for the UK and UK countries (https://jncc.gov.uk/our-work/uk-biodiversity-indicators-2023/).

## 3 Data Description

The data presented here form the basis of the categorical change metric in the State of Nature reports [6]. Data derive from two types of data – abundance and distribution (or occupancy). Abundance data at the UK level is available for 753 terrestrial and freshwater animal species. Abundance trends are based on the changes in the number of individuals at monitored sites and so reflect changes in species’ population sizes. For some taxa (e.g. bats), data on detections is collected, so the analyzed data represents local occupancy which has been shown to be a reasonable proxy for relative abundance [7]. Data for moths is based on light trap data rather than direct observation [8]. Abundance trends are only for native species and data are largely from national monitoring schemes covering the period 1970 to 2021, though periods for some species groups are shorter. Most of these datasets contained species’ time-series derived from statistical models, rather than raw counts or observations.

UK trends in distribution are available for 4979 terrestrial and freshwater invertebrate species. Distribution trends are based on changes in the number of sites where a species is present recorded at 1 km x 1 km precision (based on the British National Grid and the Irish Transverse Mercator grid). Distribution trends are primarily for native species and generally derive from biological records covering the period 1970 to 2020, though periods for some species groups are shorter. A large proportion of both types of data is collected by volunteers. Of the other taxa included in the State of Nature, trend data for vascular plants have been published [9], but data for bryophytes and fungi are not yet published.

The numbers of species with sufficient data to calculate trends declines when analyzed at the individual country level due to their smaller area, lower population levels and a range of other factors (Table 1). The number of species included in the categorical change metric for the UK differs slightly here (753; Table 1) from the report (756, page 12, [1]); two bird species with short time-series were included in error (Cattle Egret *Bubulcus ibis* and Common Redpoll *Acanthis flammea*) and Brent Goose *Branta bernicla* was included as two subspecies rather than at a specific level.

**Table 1:** Numbers of species contributing trend information to the State of Nature by country.

Taxa	Type of data	UK	England	Northern Ireland	Scotland	Wales
Terrestrial and freshwater species	Abundance	753	682	121	407	387
Terrestrial and freshwater species	Distribution	4979	4815	552	2149	3036

To gain a finer scale understanding of trends insect species are categorized into three groups that provide key ecosystem functions: pollinating insects (bees, hoverflies and moths) play a critical role in food production, predators of crop pests (ants, carabid, rove and ladybird beetles, hoverflies, dragonflies and wasps) play a key role in pest control and a range of invertebrates are involved in freshwater nutrient cycling (mayflies, caddisflies, dragonflies and stoneflies). Some species provide more than one function (e.g., adult hoverflies provide pollination services, but larvae are predators of crop pests) and so are included in more than one indicator.

## 4 Experimental Design, Materials and Methods

Trends were produced for two timescales. A long-term trend, 1970–2020/21, is effectively the longest possible run of robust data, and short-term trend, 2010–2020, to highlight recent changes in abundance or distribution. In many cases the start and/or end years for the individual species’ data did not exactly match these time periods. The abundance data ran to 2021, whilst the distribution data ran to 2020 as this is slower to become available.

### 4.1 Abundance data

Population change was described by changes in the relative abundance of species (changes in the number of individuals) and are presented in file son23_dpaper_achg_spp.csv (column headings presented in Table 2). Data were derived from a wide range of sources; details are presented in the associated data file *SoN_metadata.xlsx* (sheets 1. Change in abundance UK and 2. Abundance UK countries). Abundance trends for terrestrial and freshwater species were included if they met the following criteria: two or more methodologically consistent estimates of abundance were made between 1960 and the present and counts had adequately covered the species’ range; results, or at least the methodology for data collection and/or analysis, had been published; start and end estimates for each species were at least 10 years apart. If multiple datasets were available for a species’, the most robust dataset was used. Further details are available in Burns et al. [1,10].

**Table 2:** Column heading of the abundance trend data from *son23_dpaper_achg_spp.csv*.

scientific_name	Latin name of species from the United Kingdom Species Inventory https://www.nhm.ac.uk/our-science/data/uk-species.html
common_name	English name of species from the United Kingdom Species Inventory https://www.nhm.ac.uk/our-science/data/uk-species.html
survey	Source of data (see *Key_to_Survey_Schemes.csv* which gives the full name of each recording scheme)
group	Grouping factor used in analysis in the reporting (e.g., birds, moths)
subgroup	Second level grouping factor used in the analysis (e.g., generalist butterflies, specialist butterflies)
min_yr	First year of data availability
max_yr	Last year of data availability
annual_average_growthrate_longterm	Trend between min_yr and max_yr
change_category_longterm	Trend assessment (see Section 4.3)
annual_average_growthrate_shortterm	Trend between 2010 and max_yr
change_category_shorterm	Trend assessment (see Section 4.3)
country	UK, England, Northern Ireland, Scotland or Wales
Biome	Terrestrial and Freshwater or Marine

### 4.2 Distribution data

The terrestrial distribution time series were based on occurrence data collected by National Recording Schemes (see file *SoN_metadata.xlsx* sheet 3. Change in occupancy which lists the names of the recordings schemes providing data) and are presented in file all *invert_spp_trends.csv* (column headings in Table 3). The majority of the species’ distribution time series included in our assessment were updated versions of those presented in [11]. The trends were estimated using Bayesian hierarchical occupancy models to help control for imperfect detection [12,13]. Distribution was modelled at a 1 km x 1 km scale. Species were retained if they had a minimum of 10 years of reliable estimates and produced a distribution trend with acceptable precision [14]. These selection criteria excluded rarely recorded species and more frequently recorded species if they were only recorded in a few years. Further details in [1].

**Table 3:** Column headings for the change in occupancy data in *invert_spp_trends.csv*.

Species	Latin name of species
percent_change_year	Trend
category	Trend assessment (see Section 4.3)
time_period	lt: 1970 (or earliest data availability if later) to 2020, st: 2010 to 2020.
country	UK, England, Northern Ireland, Scotland or Wales
group	Grouping factor used in analysis in the reporting (e.g., carabids, Molluscs)
taxonomic uncertainty	Indicates if species have undergone taxonomic revision since the production of the trend data
Pollination	Insect species associated with pollination (Y or N)
Pest control	Insect species associated with pest control (Y or N)
Freshwater nutrient cycling	Insect species associated with freshwater nutrient cycling (Y or N)

Population change was described by changes in the distribution of species (changes in the number of sites where a species is found). Distribution trends were developed using a hierarchical occupancy modelling in a Bayesian framework [12,13,15]. Distribution was modelled at a 1 km × 1 km scale, with species retained if there were at least 100 records and no more than a five-year gap in records [11,15].

### 4.3 Trend assessment

A smoothed version of each species’ time-series was created using a thin plate spline model (fields::tps, [16]), with the number of knots set to a third of the time-series duration. Total change was taken as the value in the penultimate year of a smoothed species’ time-series expressed as a proportion of the first year of the assessment period. Each measure of total change was then converted to an annual average rate of change by raising it to the power of one over the duration of the assessment period and subtracting one. We placed each species into one of the five categories based upon the average annual change in relative abundance or occupancy (Table 4). All analyses were performed using R Statistical Software [17].

**Table 4:** Definitions of the categories used to classify rates of change.

Category	Rate of change	Description of change in 25 years
Strong increase	a ≥ (2^(1/25)^) − 1	Rate of change that would lead to a population doubling or more
Moderate increase	((4/3)^(1/25)^) − 1 ≤ α < (2^(1/25)^) − 1)	Rate of change that would lead to an increase of a third or more but less than doubling
Little change	(0.75^(1/25)^) − 1 < α < ((4/3)^(1/25)^) − 1	Rate of change that would lead to an increase of less than a third or a decline of less than a quarter over 25 years
Moderate decrease	(0.5^(1/25)^) − 1 < α ≤ (0.75^(1/25)^) − 1	Rate of change that would lead to a decline of greater than a quarter but less than a half over 25 years
Strong decrease	α ≤ (0.5^(1/25)^) − 1	Rate of change that would lead to a population halving or more over 25 years

## Limitations

There are a number of limitations to the data:

• There are taxonomic biases. The abundance-based trends are available for only c. 1.4 % of UK species, those typically recorded in formal monitoring schemes, and do not include important groups such as plants and fungi, as well as smaller groups such as amphibians and reptiles. There is a bias towards species easily captured in recording schemes and species that are cryptic, nocturnal, fossorial or arboreal are under-represented.
• There are geographic biases in the data as most records are from the English and Welsh lowlands, reflecting the density of recorders. The data were subject to a formal risk of bias assessment [18,19].
• The species in this dataset are those where data is sufficient to analyze, so the data is biased towards common or widespread species and taxonomic groups with greater data coverage.
• It is limited to analyzing change post-1970 and so does not include species responses to historical changes such as post-war agricultural intensification with increased mechanization, fertilizer use and pesticide applications.

## Ethics Statement

The authors have read and follow the ethical requirements for publication in Data in Brief and confirming that the current work does not involve human subjects, animal experiments, or any data collected from social media platforms.

## CRediT Author Statement

**FB:** Conceptualisation, Methodology, Formal analysis, Data curation, Writing – Review and Editing, Project administration, **SM:** Conceptualisation, Formal analysis, Data curation, Writing – Review and Editing, Project administration, **NA-F:** Data curation, Writing – Review and Editing, **PB-S:** Formal analysis, Data curation, Writing – Review and Editing, **KLB:** Formal analysis, Data curation, Writing – Review and Editing, **PB:** Data curation, **MAE:** Data curation, Writing – Review and Editing, **CH:** Formal analysis, Data curation, Writing – Review and Editing, **ACJ:** Methodology, Formal analysis, Data curation, Writing – Review and Editing, **SL:** Formal analysis, Writing – Review and Editing, **FM:** Formal analysis, Data curation, Writing – Review and Editing, **DN:** Formal analysis, Data curation, Writing – Review and Editing, **CRS:** Formal analysis, Data curation, Writing – Review and Editing, **CAS:** Data curation, Writing – Review and Editing, **RJP:** Writing original draft.

## Data Availability

ZenodoUnited Kingdom State of Nature 2023 terrestrial and freshwater animal dataset, including species trend data for England, Northern Ireland, Scotland and Wales (Original data).
